# Safety, Tolerability, and Gut Microbiota Impact of Sericin-Derived Oligopeptides (SDOs) from Yellow Silk Cocoons in Healthy Adults: A Randomized, Double-Blind, Placebo-Controlled Trial

**DOI:** 10.3390/foods15132405

**Published:** 2026-07-07

**Authors:** Sarawut Oo-puthinan, Nanteetip Limpeanchob, Watchara Pichitsiri, Apirath Wangteeraprasert, Kanittaporn Trisat, Surangkhanang Chumee, Manote Sutheerawattananonda

**Affiliations:** 1 Department of Pharmacy Practice, Faculty of Pharmaceutical Sciences, Naresuan University, Phitsanulok 65000, Thailand; sarawuto@nu.ac.th (S.O.); nanteetipl@nu.ac.th (N.L.); 2 Center of Excellence for Innovation in Chemistry, Pharmacology Research Unit, Faculty of Pharmaceutical Sciences, Naresuan University, Phitsanulok 65000, Thailand; kanittapornts@gmail.com; 3 Department of Medicine, Faculty of Medicine, Naresuan University, Phitsanulok 65000, Thailand; pichitsiri@hotmail.com (W.P.); apirathw@yahoo.com (A.W.); 4 School of Food Technology, Institute of Agricultural Technology, Suranaree University of Technology, 111 University Avenue, Muang District, Nakhon Ratchasima 30000, Thailand; palm_chumee@hotmail.com

**Keywords:** sericin-derived oligopeptides, enzymatic hydrolysis, gut microbiota, randomized controlled trial, human safety, 16S rRNA sequencing

## Abstract

Sericin-derived oligopeptides (SDOs) from the *Bombyx mori* yellow silk cocoons show strong bioactive properties. However, clinical safety data on SDOs produced by specific enzymatic hydrolysis with a particular serine-rich (20.5%) and aspartic acid-rich (16.9%) composition is required to obtain regulatory approval as a novel food ingredient. This Phase 0 randomized, double-blind, placebo-controlled trial evaluated the short-term safety, tolerability, and gut microbiota effects of SDOs supplementation in healthy adults. Forty-two healthy volunteers were randomized (1:1:1) to receive daily doses of placebo, 0.9 g SDOs or 1.8 g SDOs for eight weeks. Primary safety endpoints included vital signs, hematology, and comprehensive clinical chemistry (renal and hepatic functions). Secondary outcomes included lipid profiles, oxidative stress markers (hs-CRP, TAC, SOD, MDA) and gut microbiota composition analyzed by 16S rRNA metagenome sequencing. Forty-one participants (97.6%) completed the study with high compliance (>98%). No serious adverse events were reported. All primary clinical parameters remained within clinically normal ranges, and no significant differences between groups were observed throughout the study (*p* > 0.05). No adverse effects on fasting blood glucose, lipid profiles or systemic oxidative stress were observed after SDOs supplementation. Importantly, 16S rRNA sequencing analysis showed that SDOs maintained gut microbial homeostasis throughout the 8-week intervention period, with *Bacteroidetes* and *Firmicutes* as the predominant phyla in the core community structure. Oral intake of enzymatically generated SDOs up to 1.8 g/day in healthy adults was well-tolerated with only occasional mild and transient gastrointestinal symptoms that did not appear to be dose-dependent. These first preliminary findings suggest a favorable safety profile for this unique peptide preparation, supporting its potential evaluation as a novel food ingredient and providing a reasonable basis for future, larger-scale trials to evaluate its efficacy in metabolic health.

## 1. Introduction

The increasing occurrence of non-communicable diseases at a global level, particularly those related to metabolic dysfunction such as hypertension, dyslipidemia, and type 2 diabetes, constitutes a major public health problem. These conditions are the primary contributors to cardiovascular disease, the world’s leading cause of death. Despite drugs being the primary treatment option, their limitations, such as adverse effects, poor patient adherence and high health care costs, have promoted a trend towards more holistic prevention and treatment strategies. As a consequence, there is increasing interest in functional foods and nutraceuticals, particularly bioactive peptides derived from natural proteins. These peptides are known for their multi-target action and safety and are considered a promising tool for sustainable health management.

The sericulture industry is a unique and historically underutilized resource. Sericin is a globular protein, which constitutes approximately 30–35% of the *Bombyx mori* silk cocoon, and is traditionally solubilized and discarded as an effluent during the silk reeling process. However, this industrial waste is now being considered as a source of a variety of useful bioactive compounds. Studies have demonstrated that sericin exhibits a variety of pharmacological activities such as strong antioxidant activity, inhibition of colon carcinogenesis, favorable modulation of serum lipids [1,2,3,4,5,6,7], prevention of alcohol-induced liver injury, antiviral effect and hypotensive activity [8,9,10,11].

In native form, sericin is a large protein polymer with a molecular weight of 20–400 kDa, and is often hydrolysed to enhance its biological utility. Hydrolysis breaks down the large protein into smaller, water-soluble SDOs. This conversion is well-founded as smaller peptides are generally better absorbed in the intestine and are more bioavailable than larger parent proteins. Smaller fractions of sericin (5–100 kDa) have been shown to be more effective in stimulating mammalian cell proliferation than larger fractions [12,13,14]. Development of SDOs is based on this principle with the hypothesis that they will possess better biological efficacy.

SDOs have been shown to significantly improve blood cholesterol profiles in rats fed high-cholesterol or high-fat diets [4]. Moreover, their capacity to exert both antihypertensive and antidiabetic effects in various rodent models highlights their multi-target potential [11,15]. Crucially, the safety profile of SDOs was rigorously established prior to any human investigation. In accordance with OECD standard protocols, comprehensive toxicological assessments in Wistar rats and ICR mice revealed no acute toxicity at a single high dose of 2000 mg/kg BW [16]. Furthermore, chronic six-month administration at doses up to 200 mg/kg BW produced no pathological abnormalities. Moreover, a recent study has demonstrated that SDOs are effective cognitive-enhancing agents that reduce memory deficits in models of acute neurotoxicity and chronic aging [17].

Although preclinical results are encouraging, there is a significant barrier to the translation of these results to human use. Other products referred to as “silk peptides” have been studied in previous clinical trials, but they should be differentiated from the SDOs used in the present study because they are fundamentally different in both composition and production, as presented in Table 1 [18]. The previously studied peptides were derived from a mixture of fibroin and sericin by intensive acid hydrolysis, yielding a product with a different amino acid profile comprising a high proportion of glycine (31.3%) and alanine (20.0%). By contrast, the SDOs studied here are produced by controlled, food-grade enzymatic hydrolysis of pure sericin extracted from yellow silk cocoons [19,20,21,22,23]. This process results in a unique compositional fingerprint with high levels of serine (20.5%) and aspartic acid (16.9%). Accordingly, safety data from previous research using acid-hydrolyzed mixed silk peptides cannot be directly applied to these unique, enzymatically derived SDOs. This distinction is important because the controlled enzymatic process preserves specific peptide sequences and bioactivities that would otherwise be destroyed by harsh chemical treatments, necessitating a separate clinical evaluation of this particular preparation. To date, there is a major dearth of published clinical data specifically examining the safety and tolerability of these particular SDOs for human consumption.

Beyond systemic safety, the interaction of bioactive peptides with the gastrointestinal environment must be thoroughly explored. The gut microbiota is crucial for host metabolism and immune function, and any novel dietary protein must be assessed for its potential to perturb this delicate ecosystem. SDOs are the major interfaces with intestinal bacteria; it is important to verify that their particular amino acid composition (particularly the high serine and aspartic acid content) does not lead to dysbiosis or unfavorable alterations in the microbial community structure. Establishing the “microbial safety” of SDOs is therefore a key component of their evaluation as a sustainable and safe novel food ingredient.

To close this critical gap and meet stringent regulatory requirements for novel food ingredients, a formal human safety study is required. We hypothesized that short-term oral supplementation of these unique SDOs would be safe and well-tolerated in healthy individuals without perturbing metabolic homeostasis or causing adverse changes in key physiological and biochemical markers. We therefore undertook a rigorous randomized, double-blind, placebo-controlled trial to formally assess the safety and tolerability of SDOs. Using a healthy cohort, the aim of this study is to establish a rigorous safety baseline, ensuring that SDOs do not interfere with normal glucose, lipid metabolism, and the composition of the gut microbiome prior to future efficacy trials in clinical populations.

## 2. Materials and Methods

### 2.1. SDOs Preparation

#### 2.1.1. Preparation and Production of SDOs Powder

The sericin concentrate was provided by Thai Silk Products Co. Ltd. (Nakhon Ratchasima, Thailand). It was prepared by repeated extraction of native yellow silk cocoon shells (*Bombyx mori*, Nang Noi Srisaket-1 variety) in deionized (DI) water at 95 °C in a double boiler until the cocoon shells were off-white. The collected sericin solution was concentrated by using a pan extractor to have a final solid concentration of 20% (*w*/*v*). This concentrate was stored at −20 °C until use.

Enzymatic hydrolysis was performed at the Fermentation Technology Research and Service Center (FTC), Kasetsart University (Bangkok, Thailand), following a previously described method in Sangsawad et al. (2025) [24]. Sericin concentrate was reconstituted with DI water to 15% (*w*/*v*). Hydrolysis was initiated by adding Protease N (Brenntag Ingredients Co., Ltd., Bangkok, Thailand) at an enzyme-to-substrate ratio (E/S) of 5%. The mixture was incubated at 55 °C, pH 7.0, for 4 h in a 300 L tank. The resulting SDOs solution was filtered through sterilized cheesecloth, cooled and then spray-dried at an inlet temperature of 180 °C using a SPRAY DRYER SDG-100 NF (Euro Best Company Co. Ltd., Pathum Thani, Thailand) to obtain the final SDOs powder.

#### 2.1.2. Physicochemical Characterization and Safety Analysis of SDOs Powder

The final SDOs powder was analyzed prior to the clinical trial for quality and safety. Protein content was determined using the Kjeldahl method [25,26], and the amino acid composition was determined using an in-house AOAC method 982.30 [27]. Bioactivity was confirmed by dipeptidyl peptidase-IV (DPP-IV) and angiotensin-converting enzyme (ACE) inhibitory activities [28,29].

The safety analysis was performed by Central Laboratory (Thailand) Co., Ltd. (Khon Kaen, Thailand) according to the standards of the Thai FDA. Heavy metals (As, Cd, Pb, Hg) were tested according to EPA 3052 [30]. Pesticide residues (carbamate, organochlorine, organophosphate, pyrethroid) were analyzed by the in-house QuEChERS method (EN 15662:2018) [31]. Biological contaminants (*Escherichia coli*, *Listeria monocytogenes*, *Salmonella* spp., *Staphylococcus aureus*, total plate count, yeasts/molds) were determined using the FDA Bacteriological Analytical Manual (BAM) or ISO standard methods indicated [32,33,34,35,36,37].

#### 2.1.3. Preparation of Study Products (Capsules) and Blinding

The investigational products were prepared as hard capsules. The active capsules contained 225 mg of SDOs powder. The placebo capsules contained 225 mg maltodextrin DE 10 (Chemipan Corporation Co., Ltd., Bangkok, Thailand) as the placebo. All hard gelatin capsules were provided by Vechakit Chemical Co., Ltd. (Bangkok, Thailand). To maintain the double-blind design, the active and placebo capsules were identical in appearance, size, color and odor. Quality control analyses performed prior to distribution confirmed that the encapsulated products met the standards of the United States Pharmacopeia (USP) XX. Weight variation was between −2.37% and +2.42% (within the required ±7.5% limit), and disintegration time was between 15 and 25 min (well within the 30 min requirement).

#### 2.1.4. Stability Testing

A 6-month stability study was performed to determine optimal storage conditions for the clinical trial and to estimate the shelf-life of the SDOs powder. Samples of SDOs powder were stored under three different conditions: 4 °C, 25 °C and 45 °C. At predetermined time points, samples were analyzed for key stability-indicating parameters, including moisture content [26], water activity (aW; Aqua Lab series 3 TE, Decagon Devices, Inc., Pullman, WA, USA), color (CIE Lab*; ColorQuest XE, HunterLab, Reston, VA, USA) and the IC50 of DPP-IV and ACE inhibitory activities.

### 2.2. Study Design and Ethical Approval

This investigation was conducted as a randomized, double-blind, placebo-controlled, parallel-group exploratory trial to assess the safety and tolerability of two different daily doses of SDOs over an 8-week period. The study protocol and all related documents received approval from the Institutional Review Board of Naresuan University in Phitsanulok, Thailand (IRB no: 483/58). The Thai Clinical Trial Registration number TCTR20240618003 is available at https://www.thaiclinicaltrials.org.

The trial was conducted in full accordance with the principles of the Declaration of Helsinki and the guidelines for Good Clinical Practice (GCP). All participants provided written informed consent before any study-related procedures were performed.

### 2.3. Dose Rationale and Sample Size Justification

The doses chosen for evaluation were supported by a large body of preclinical data. The No Observed Adverse Effect Level (NOAEL) in chronic toxicity studies in rodents was 2 g/kg/day, representing a large margin of safety. At the same time, doses predicted to elicit a therapeutic cholesterol-lowering effect in rats (10–200 mg/kg/day) were translated into a human equivalent dose range of approximately 100–2000 mg/day for a 60 kg individual. Using this combined safety and efficacy data, two doses—900 mg/day and 1800 mg/day—were chosen to assess the safety profile within this projected therapeutic window.

Given that the primary objective of this study was a preliminary assessment of safety and tolerability in a pilot cohort, a formal statistical power calculation for sample size was not undertaken. Instead, the sample size was selected pragmatically based on current best practice for early-phase safety trials and regulatory guidance. This sample size is consistent with prior recommendations for pilot safety and tolerability trials and will provide sufficient data to identify common adverse events and establish a baseline for future larger-scale efficacy studies [38,39,40]. We planned to enroll 14 participants per group, which, allowing for an anticipated dropout rate of approximately 15%, would ensure at least 12 participants per arm completed the study. This resulted in a total target enrolment of 42 participants, which is considered sufficient to identify common adverse events and provide a preliminary assessment of the safety profile of SDOs.

### 2.4. Participants

**Figure 1:**
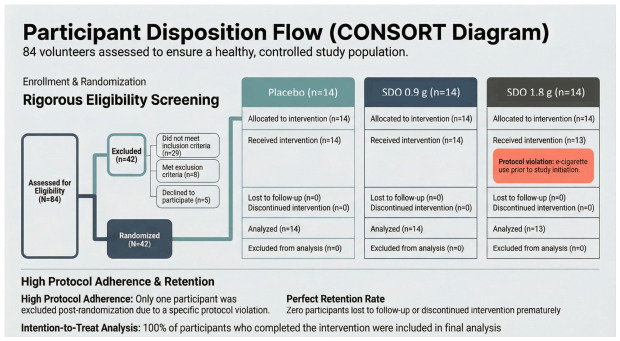
CONSORT flow diagram of participant disposition throughout the trial. A total of 84 volunteers were screened for eligibility, of whom 42 were randomized into three groups (placebo, SDOs900, and SDOs1800; n = 14 per group). One participant in the SDOs1800 group was excluded post-randomization for failing to follow study instructions prior to receiving the first intervention. All remaining 41 participants completed the 8-week study and were included in the final analysis.

Healthy male and female volunteers aged 20 to 45 years with a body mass index (BMI) ranging from 18.5 to 25.0 kg/m^2^ and no history of protein allergies were recruited from the community in Phitsanulok, Thailand. (1) The participant screening and enrollment process is shown in the CONSORT flow diagram (Figure 1). (2) A total of 84 volunteers were screened initially, among whom 50 were invited for a comprehensive screening visit, including a physical examination, medical history review, and laboratory screening tests. After this rigorous process, 42 eligible participants were enrolled and randomized in a 1:1:1 ratio into three parallel groups using a block randomization method. An independent researcher generated randomization sequences using a computer-based block randomization method. The allocation sequence was maintained in sequentially numbered, opaque, sealed envelopes and was maintained by an independent researcher, who dispensed the investigational products to achieve double blinding of investigators and participants. (3) The groups were: placebo, SDOs900 (900 mg/day SDOs), and SDOs1800 (1800 mg/day SDOs). Participants were instructed to take eight capsules daily for eight weeks in two doses. (4) The SDOs900 group took 4 active capsules before breakfast and 4 placebo capsules before bedtime, while the SDOs1800 group took 4 active capsules at both time points. All participants followed the same twice-daily regimen to maintain blinding. (5) Follow-up visits were conducted in weeks 4 and 8 for physical examinations, biochemical blood tests, urine analysis, and adverse event monitoring. Each subject’s feces were also collected before the beginning (week 0) and the end of the study (week 8) to investigate any changes in gut microbiota. Adherence to the study protocol was assessed at each follow-up visit by counting returned capsules and reviewing participant self-recorded logs. Participants with a compliance rate of less than 85% (calculated by returned capsule counts) were predefined to be excluded from the per-protocol analysis. Throughout the 8-week study period, all participants were explicitly instructed to maintain their habitual dietary patterns, physical activity levels, and lifestyle habits to minimize confounding variables.

### 2.5. Study Outcome Measurements

All outcomes were measured at baseline (week 0) and at follow-up visits on weeks 4 and 8. The primary outcomes of this study were the assessment of safety and tolerability. Safety was evaluated by the incidence of adverse events, physical examinations including vital signs (blood pressure and heart rate), and comprehensive laboratory safety data including complete blood count (CBC), urinalysis (pH, specific gravity, glucose, protein, and cells), and clinical chemistry panels for renal (BUN, creatinine) and liver function (AST, ALT, ALP, albumin, bilirubin). Secondary and exploratory outcomes were also assessed, including the participant’s lipid profile (total cholesterol, LDL-C, HDL-C, and triglycerides), a marker of inflammation (hs-CRP), and markers of oxidative stress. The latter were total antioxidant capacity (TAC), superoxide dismutase (SOD) activity, and malondialdehyde (MDA). All routine clinical parameters (hematology, clinical chemistry, lipid profile and hs-CRP) were analyzed at the accredited clinical laboratory of Naresuan University Hospital (Phitsanulok, Thailand) following ISO 15189:2012 [41] and ISO 15190:2020 [42]. TAC and SOD activities, and MDA were determined by commercially available assay kits (TAC kit, Lot No. GR3306683-3; SOD activity kit, Lot No. GR3318066-1; MDA assay kit (competitive ELISA) ab238537, Lot No. GR3318035-1, Abcam, Cambridge, MA, USA) according to the manufacturer’s instructions.

For the assessment of gut microbiota composition, stool nucleic acid collection and preservation tubes were purchased from Norgen Biotek, Thorold, ON, Canada (Lot No. 595686). Gut microbiota analysis of fecal samples was performed by Macrogen Inc. (Seoul, Republic of Korea) using 16S rRNA gene amplicon sequencing. The V3–V4 region of the 16S ribosomal RNA was amplified using primers with Illumina overhang adapter sequences (Illumina, San Diego, CA, USA). The primer sequences were as follows: forward primer 5′-TCGTCGGCAGCGTCAGATGTGTATAAGAGACAG-3′ and reverse primer 5′-GTCTCGTGGGCTCGGAGATGTGTATAAGAGACAG-3′. PCR products were purified, followed by a second PCR to attach dual indices and sequencing adapters. The indexed libraries were purified, quantified, normalized, and pooled prior to paired-end sequencing on an Illumina MiSeq platform according to Macrogen’s standard protocol. Filtered reads were clustered at 100% identity using CD-HIT-DUP. Remaining representative reads from non-chimeric clusters were clustered into operational taxonomic units (OTUs) at 97% sequence identity using the q2-vsearch plugin within the QIIME 2 pipeline. Taxonomic classification was assigned against the NCBI 16S rRNA database, up to the species level. OTUs from all samples were filtered prior to alpha diversity, beta diversity, and differential abundance analyses. Specifically, OTUs were retained only if they met two criteria: having at least 3 read counts in a minimum of 5% of the samples, and representing at least 0.01% of the total sequence read counts. Following filtering, the dataset was rarefied to a depth equivalent to the minimum total read counts per sample for diversity analyses. Alpha diversity indices, including observed OTUs, Chao1, Shannon index and Inverse Simpson index, and global beta diversity metrics across all experimental conditions, including Bray–Curtis dissimilarity, Jaccard distance and Aitchison distance, were determined using the vegan package (version 2.6-10).

### 2.6. Statistical Analysis

Before applying the parametric tests, normality of continuous data distribution was tested with the Shapiro–Wilk test and homogeneity of variances was tested with Levene’s test. The baseline characteristics were compared using one-way ANOVA or Fisher’s exact test. A *p*-value of ≤0.05 was considered statistically significant. The differences in the continuous outcome variables between the groups (placebo, SDOs 0.9 g, and SDOs 1.8 g) over time points were statistically compared using a linear mixed-effects model (LMM). The LMM incorporated Group, Time (week 4 and week 8) and their interaction (Group × Time) as fixed factors. Baseline values (week 0) were included as a covariate in the model to control for differences at the beginning. A random intercept was assigned to each subject to control for inter-individual variability, and parameters were estimated by the Restricted Maximum Likelihood (REML) method. When main or interaction effects were statistically significant, post hoc pairwise comparisons were conducted using the Sidak adjustment to control for multiple comparisons. All routine statistical analyses were performed using SPSS version 21 (IBM Corp., Armonk, NY, USA).

Gut microbiome data analyses were performed in R Studio (version 2025.05.1 Build 513) and R (version 4.5.0). Nonparametric analysis of longitudinal data (nparLD) was used to compare changes over time in alpha diversity indices between the groups with the nparLD package (version 2.2) [43].To compare changes over time in global beta diversity metrics among the groups, a permutation-based multivariate analysis of variance (PERMANOVA) was performed with 9999 permutations using the adonis2 function of the vegan package (version 2.6-10) [44,45]. Changes in differential abundances over time between groups at the family, genus and species levels were assessed using ANCOM-BC2 (compositions of microbiomes with bias correction 2) with 100 permutations using the ANCOMBC package (version 2.10.1) [46]. Group and time were fixed effects, and the within-subject factor was the random effect in nparLD, PERMANOVA and ANCOM-BC2 analysis. The false discovery rate was controlled at 5% significance level using the Benjamini–Hochberg (BH) method for multiple comparisons.

## 3. Results

### 3.1. Characterization and Safety Profile of SDOs Powder

The SDOs powder prepared at 300 L pilot scale for clinical trial use was first characterized in terms of its physicochemical properties, bioactivity and safety. The production process led to high solid and protein recoveries of 94.35 ± 0.70% and 91.23 ± 0.23%, respectively, and a final degree of hydrolysis of 8.63 ± 0.92%. The obtained SDOs powder showed potent bioactivity, showing 49.83 ± 1.59% inhibition of dipeptidyl peptidase-IV (DPP-IV) and 65.83 ± 0.59% inhibition of angiotensin-converting enzyme (ACE) under the assay conditions tested.

Importantly, the SDOs powder was shown to be safe for human consumption before its use in this study (Table 2). The concentrations of all heavy metals and microbial contaminants analyzed were well below the legal limits set by the Thai Food and Drug Administration (FDA). Arsenic (As), lead (Pb) and mercury (Hg) levels were minimal, and cadmium (Cd) was not detectable. Microbiological testing also confirmed the absence of pathogenic bacteria such as *Listeria monocytogenes* and *Salmonella* spp. Furthermore, all pesticide residues were undetectable.

### 3.2. Amino Acid Composition of SDOs Powder

Table 3 shows the amino acid composition of SDOs powder used in the clinical trial. The results showed a specific amino acid profile with a high content of serine (20.50 g/100 g) and aspartic acid (16.89 g/100 g). Other significant amino acids were glycine (5.22 g/100 g), threonine (5.20 g/100 g) and lysine (5.01 g/100 g). The total content of the quantified amino acids was 79.40 g/100 g of dry powder. Consistent with other sericin hydrolysates, asparagine and tryptophan were not detected in the analysis.

### 3.3. Stability of SDOs Powder over 6 Months

To ensure the quality and consistency of the investigational product throughout the clinical trial, a 6-month stability study of the SDOs powder was conducted under three storage conditions (4 °C, 25 °C, and 45 °C).

#### 3.3.1. Physical Stability

The physical stability of the SDOs powder was analyzed by tracking moisture content, water activity (aW), color, and solubility (Table 4, Table 5 and Table 6). Moisture content and aW were stable during the first three months in all the conditions. At the time point of 6 months, however, both parameters showed a minor but statistically significant decrease (*p* < 0.05) (Table 4). Despite these changes, the aW value was always below 0.5, indicating a low risk of microbial degradation.

Color stability was temperature dependent (Table 5). All storage temperatures showed a significant increase in total color difference (ΔE) over time, with the most drastic changes at 45 °C. The ΔE value at 45 °C was already above 5 (ΔE = 5.15) after 3 months, indicating a significant color difference, and further increased to 10.41 at 6 months.

Particle size distribution did not change during 6 months of storage at all temperature conditions, and there was no significant difference in mean particle diameter (*p* > 0.05) (Table 6). The temperature also affected solubility. At 4 °C, there was no significant change in solubility, but at 25 °C, a slight decrease of about 3% was observed after 6 months. At 45 °C, solubility decreased significantly by about 7% from the initial value (*p* < 0.05) (Table 6).

#### 3.3.2. Bioactivity and Chemical Stability

The stability of the bioactivity of SDOs powder was evaluated by measuring its DPP-IV and ACE inhibitory activities (Table 7). The ACE inhibitory activity (IC50) was very stable and showed no significant difference from the baseline after storage for 6 months at 4 °C and 25 °C (*p* > 0.05). Only at 45 °C after 6 months, a small but statistically significant increase in the IC50 value (corresponding to a small loss of activity) was observed.

Conversely, the DPP-IV inhibitory activity was less stable. A statistically significant increase in IC50 value was observed at the 6-month time point for all three storage conditions (*p* < 0.05). The loss of inhibitory efficiency was most pronounced at 45 °C, where the IC50 value increased by 39.94% compared to 27.06% at 25 °C and 25.77% at 4 °C.

**Figure 2:**
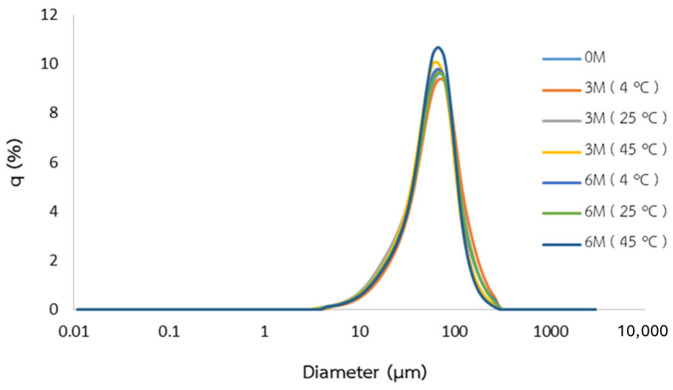
Particle size distribution of SDOs powder at baseline (0M, 0 month) and after 3 and 6 months of storage at different temperatures. The overlay of the distribution curves shows that particle size remained highly consistent across all tested conditions and time points, with a primary peak centered around 80–100 µm.

The molecular weight distribution of the peptide fractions was rather stable despite the observed changes in some of the physicochemical parameters and DPP-IV activity. As can be seen in Figure 2, the molecular weight distribution curves obtained from all storage conditions and time points overlapped almost perfectly, indicating that no significant peptide aggregation or degradation occurred during the 6-month storage period.

From these extensive stability data, and particularly the better preservation of bioactivity, it was decided that the SDOs powder should be kept at 4 °C throughout the human clinical trial.

### 3.4. Participant Characteristics and Disposition

Having established the physicochemical stability, bioactivity, and safety of the SDOs powder, we proceeded to evaluate its tolerability in the human clinical trial. A total of 42 healthy volunteers were randomized into the study. The baseline demographic and physical characteristics of the participants are presented in Table 8. The study population was predominantly female (75.6%), with a mean age of 30.6 ± 6.6 years and a mean BMI of 21.7 ± 2.0 kg/m^2^. There were no statistically significant differences among the placebo, SDOs 0.9 g, and SDOs 1.8 g groups at baseline for any of the recorded characteristics, including sex, age, BMI, height, and weight (*p* > 0.05 for all), indicating that the randomization process was successful.

Participant disposition throughout the trial is detailed in the CONSORT flow diagram (Figure 1). One participant randomized to the SDOs 1.8 g group was excluded prior to the first intervention for failing to follow study instructions. All remaining 41 participants completed the 8-week study protocol. Adherence to the study intervention was excellent across all groups, with an average compliance rate exceeding 98%. The lowest individual compliance rate recorded was 87%, confirming that all participants included in the analysis had a high level of adherence.

### 3.5. Preliminary Safety on Clinical Parameters

The 8-week oral administration of sericin-derived oligopeptides (SDOs) at doses reaching 1.8 g/day demonstrates a preliminary safety profile, maintaining systemic physiological homeostasis without evidence of treatment-induced perturbation. 

Vital Signs and Renal and Hepatic function (Table 9): Cardiovascular parameters remained within normal clinical ranges throughout the study duration. While a significant group × time interaction was observed for heart rate (*p* = 0.023), post hoc analysis confirmed this was isolated to fluctuations within the placebo group (*p* = 0.010) rather than a treatment effect. Comprehensive biochemical analysis confirmed the preservation of renal and hepatic integrity. Renal markers, including creatinine and blood urea nitrogen, showed no significant deviations. Urine analyses were clinically unremarkable between the experiment and placebo groups. Liver function markers, including total bilirubin, AST, ALT, and ALP, remain consistently within normal clinical ranges across all groups, indicating no hepatotoxic or nephrotoxic effects.

Hematology and Metabolic Markers (Table 10): Hematological stability was similarly preserved, with all primary markers (WBC, RBC, Hb, and Platelets) remaining stable (*p* > 0.05). Although a statistical interaction was noted for hematocrit (*p* = 0.035), specifically due to a variation between weeks 4 and 8 in the 0.9 g group (*p* = 0.007), all values remained strictly within standard clinical limits, indicating no adverse impact on hematopoietic function. Metabolic homeostasis was maintained across all experimental arms. While a significant main effect of time was observed (*p* = 0.003) for fasting blood glucose (FBS) levels, this represents normal physiological variation as all mean values remained normoglycemic. Lipid profiles, including total cholesterol, LDL-C, and HDL-C, remained stable. High variability observed in triglyceride levels within the SDOs 1.8 g group (baseline: 105.4 ± 94.2 mg/dL) was attributed to a specific participant outlier with baseline levels exceeding 300 mg/dL, rather than a systematic response to SDOs supplementation.

### 3.6. Effects on Markers of Oxidative Stress and Inflammation

Systemic Oxidative Stress and Inflammation (Table 11): SDOs supplementation did not trigger systemic inflammatory or oxidative stress responses. Markers, including hs-CRP, TAC, SOD, and MDA, followed consistent patterns across all treatment and placebo groups. For TAC, SOD, and MDA, significant main effects of time were observed (*p* < 0.01), yet the absence of group differences confirms that these fluctuations represent cohort-wide temporal variations rather than a specific response to SDOs ingestion.

### 3.7. Adverse Events and Tolerability

All treatment-emergent adverse events (TEAEs) reported during the 8-week study period are shown in Table 12. Overall, SDOs supplementation was well-tolerated. There were no serious adverse events (SAEs) or deaths during the study, and no participants withdrew from the trial due to adverse events.

Headache was the most commonly reported TEAE in all groups. However, a clear causal relationship with the SDOs product could not be established, as the SDOs 0.9 group reported fewer cases than the placebo group, whereas the SDOs 1.8 group showed a higher incidence. Gastrointestinal symptoms were also common. Three participants (21.4%) in the SDOs 0.9 g group and two participants (15.4%) in the SDOs 1.8 g group reported mild diarrhea, whereas none were reported in the placebo group. However, no clear dose–response relationship was observed. Other common symptoms, such as bloating, rhinorrhea and constipation, occurred at similar frequencies between the placebo and SDOs groups, suggesting that they were unlikely to be related to the study product. All reported adverse events were mild to moderate in severity and resolved without sequelae.

### 3.8. Effects of SDOs on Gut Microbiota

After establishing the systemic safety and tolerability of SDOs, we further investigated their effect on the gastrointestinal ecosystem through 16S rRNA metagenome sequencing. The fecal microbiota profiles of the study participants were analyzed by 16S rRNA metagenome sequencing. The minimum and maximum total read counts in the samples were 4019 and 17,886, respectively. Good’s coverages were calculated for all samples, with a median of 99.79% (range: 99.14–99.97%), indicating high sequencing completeness. The median total read counts, together with the interquartile range (IQR), for the placebo, SDOs 0.9 g, and SDOs 1.8 g groups before the study (week 0) were 10,495 (3883), 11,320 (3289), and 10,495 (3365), respectively. At the end of the study (week 8), the median total read counts were 6646 (2864), 7151 (1993), and 7103 (3421), respectively. These median total read counts were found to be comparable across the different groups.

**Figure 3:**
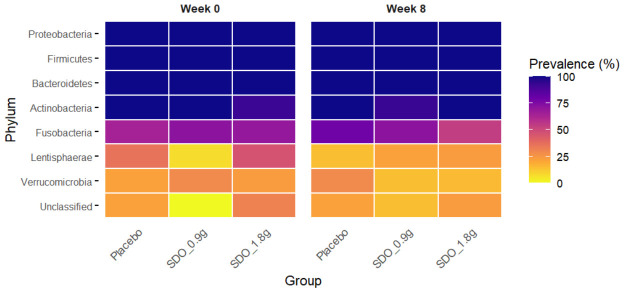
Prevalence heatmap of individual phylum by group at week 0 and week 8.

**Figure 4:**
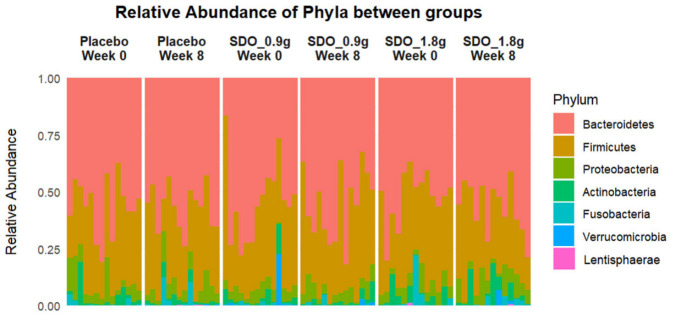
Relative abundance of gut bacteria at the phylum level in three experiment groups at the start of the study (week 0) and at week 8.

Seven phyla of gut bacteria were identified in this study. The core bacterial phyla, defined as a minimum 90% prevalence, were Bacteroidetes, Firmicutes, Proteobacteria and Actinobacteria (Figure 3). It should be noted that the first three phyla were detected in all subjects. Bacteroidetes and Firmicutes were the most dominant bacterial phyla, followed by Proteobacteria and Actinobacteria, with median relative abundances of approximately 50–56%, 31–38%, 5–6% and 1%, respectively, in all three experimental groups (Figure 4 and Table 13). The most common bacterial groups were Bacteroidia, Clostridia and Negativicutes with median relative abundances of 50–56%, 21–23% and 6–8%, respectively. At the family level, Bacteriodaceae was the most relatively abundant family, with the median relative abundance ranging from 21 to 32%, followed by Oscillospiraceae and Lachnospiraceae. The top seven genera were *Phocaeicola*, *Bacteroides*, *Faecalibacterium*, *Phascolarctobacterium*, *Prevotella*, *Alistipes* and *Bifidobacterium*, with *Phocaeicola* being the major genus at 11–21% relative abundance. A total of 198 OTUs were found in this study. A total of 22 core species (defined as having an overall prevalence ≥ 90%) were identified: *Bacteroides thetaiotaomicron*, *Blautia luti*, *Enterocloster clostridioformis*, *Kineothrix alysoides*, *Phocaeicola vulgatus*, *Blautia wexlerae*, *Streptococcus salivarius*, *Bacteroides uniformis*, *Parabacteroides distasonis*, *Escherichia fergusonii*, [*Eubacterium*] *rectale*, *Bacteroides caccae*, and *Oscillibacter ruminantium*. *Faecalibacterium prausnitzii*, *Flintibacter butyricus*, *Bacteroides kribbi*, *Collinsella aerofaciens*, [*Ruminococcus*] *torques*, *Anaerobutyricum hallii*, *Blautia faecis*, *Parabacteroides merdae*, and *Bifidobacterium adolescentis*. As shown in Table 14, *Phocaeicola vulgatus* was the most abundant species, with relative abundance ranging from 6 to 13%.

**Figure 5:**
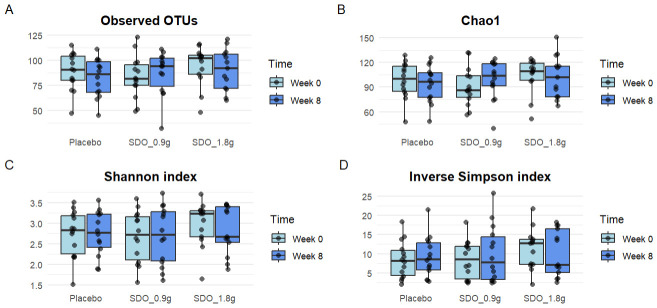
Box plots of alpha diversity indices, including (**A**) observed OTUs; (**B**) Chao1; (**C**) Shannon index; and (**D**) Inverse Simpson index of placebo, SDOs 0.9 g/day, and SDOs 1.8 g/day groups at week 0 and week 8.

The richness and evenness of gut microbiota were determined using alpha diversity indices, including observed OTUs, Chao1, Shannon index and Inverse Simpson index as shown in Table 15 and Figure 5. For Observed OTUs and Chao1, there were significant interaction effects between Group and Time (*p* = 0.025 and 0.039, respectively). This indicated that the changes in microbial richness over time were not consistent across the placebo, SDOs 0.9 g/day, and SDOs 1.8 g/day groups. However, the 95% confidence intervals for the Relative Treatment Effects (RTEs) for all individual group–time combinations contain 0.5, indicating that no single group at any time point significantly deviated from the overall average richness. There were no significant differences between all three groups for Shannon metrics and Inverse Simpson metrics.

**Figure 6:**
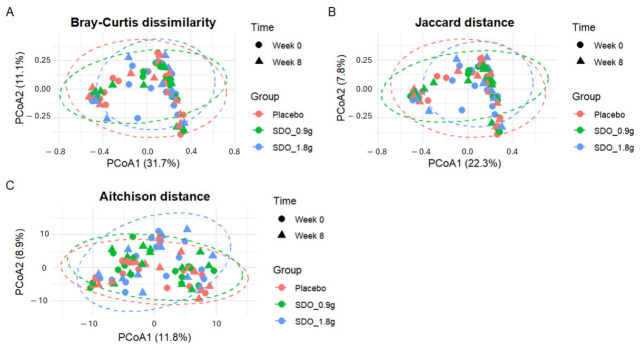
PCoA plot for beta diversity metrics across groups including (**A**) Bray-Curtis dissimilarity; (**B**) Jaccard distance; (**C**) Aitchison distance at week 0 and week 8.

Principal coordinate analysis (PCoA) plots of overall beta diversity metrics (Bray–Curtis dissimilarity, Jaccard distance and Aitchison distance) by group at week 0 and week 8 are shown in Figure 6, with no clear differences observed for bacterial community composition by group or time point. PERMANOVA analysis, however, did reveal a statistically significant difference between groups for the Aitchison distance metric (*p* = 0.041), although no significant pairwise differences were observed post hoc.

No differentially abundant taxa were observed with ANCOM-BC2 differential abundance analysis at the family, genus or species levels for either study groups or time points. Together, these high-resolution sequencing data indicate that 8-week supplementation of SDOs maintained microbial homeostasis without significantly altering the core microbial community structure in healthy adults.

## 4. Discussion

This study is the first exploratory Phase 0 clinical evaluation of the safety and tolerability of enzymatically produced SDOs from yellow silk cocoons in healthy human subjects. Findings from this randomized, double-blind, placebo-controlled trial show that oral supplementation with SDOs at doses up to 1.8 g/day for 8 weeks was well-tolerated with no serious adverse events reported.

The most commonly reported adverse event possibly related to SDOs was mild, transient diarrhea (21.4% in the 0.9 g group and 15.4% in the 1.8 g group); these incidents were not dose-dependent. Mild diarrhea, not reported in a similar 8-week study by Hwang et al. on a different silk peptide product, should be considered. This difference may be due to the fundamental differences between the products; the SDOs in our study are a unique preparation of pure sericin hydrolysate that is rich in serine and aspartic acid, whereas the product in the Hwang et al. (2019)’s study was an acid-hydrolyzed mixture of fibroin and sericin, rich in glycine and alanine [18]. The reported mild diarrhea was transient and did not lead to any study withdrawals, suggesting a minor osmotic effect or gut adaptation to concentrated peptides rather than systemic toxicity, leading to mild intraluminal water retention in the bowel—a phenomenon frequently observed with highly concentrated protein or peptide supplements when intestinal peptide transporters (e.g., PepT1) are temporarily saturated [47]. Importantly, all cases are resolved spontaneously. The lack of dose-dependency in diarrhea incidence (SDOs 0.9 g vs. SDOs 1.8 g) further supports a threshold-based physiological response rather than a cumulative toxic effect. This is in agreement with the high compliance rate (>98%) observed. Other common gastrointestinal symptoms, such as bloating (14.3% in placebo vs. 35.7% in SDOs 0.9 g or 7.7% in SDOs 1.8 g) and constipation (14.3% in placebo vs. 14.3% in SDOs 0.9 g), occurred at comparable frequencies across groups, suggesting they were unlikely to be causally related to SDOs consumption.

Following potential absorption, systemic safety of SDOs was extensively evaluated by vital signs, complete blood count (CBC) and routine liver and kidney function tests. Crucially, no parameters demonstrated clinically significant changes. Although one participant in the SDOs 1.8 g group exhibited a transient elevation in ALT to 104 U/L at week 8 (compared to 34 U/L at week 0 and 18 U/L at week 4), clinical review revealed this individual had received a third COVID-19 vaccine dose and taken paracetamol for post-vaccination pain three days prior to the visit; this value returned to the normal range during post-study follow-up without associated clinical symptoms. Similarly, a participant in the placebo group experienced a transient ALT elevation at week 8 (49, 44, and 90 U/L at weeks 0, 4, and 8, respectively) after also recently receiving a COVID-19 vaccination and paracetamol. Given that AST levels remained within the acceptable range across all experimental groups, it remains unclear whether these isolated, transient ALT elevations were related to the SDOs product, vaccination, or paracetamol use [48].

Given the healthy, normoglycemic, and normolipidemic profiles of the population in this study, the ability to observe any potential effects of SDOs on metabolic homeostasis was inherently limited. In line with this, FBS and lipid profiles (TC, LDL-C, HDL-C, TG) showed no statistically significant differences between the SDOs and placebo groups (Table 10). The stability of these parameters in a healthy population supports the metabolic tolerability of SDOs, confirming that they do not induce hypoglycemia or detrimental changes in lipid profiles under normal physiological conditions. While preclinical studies demonstrated cholesterol-lowering and hypoglycemic effects in models of metabolic compromise [4,15], the current results are intended to establish a safety baseline in normoglycemic and normolipidemic individuals rather than evaluate metabolic efficacy. Similarly, the evaluation of antioxidants and anti-inflammatory effects was likely constrained by the healthy baseline status of the participants. Parallel to the metabolic findings, markers of systemic inflammation (hs-CRP), oxidative stress (MDA), and antioxidant activity (TAC, SOD) showed no statistically significant differences between groups (Table 11).

The potential of SDOs as a novel food ingredient is supported by their high bioavailability and stability during digestion. Crucially, SDOs exhibit significant resistance to further enzymatic degradation within the gastrointestinal tract. Recent peptidomic characterization of this specific SDOs preparation identified 32 unique sequences, many of which contain C-terminal proline residues, a structural motif known to protect peptides from breakdown by digestive proteases [21]. Simulated gastrointestinal digestion models have confirmed that these peptides remain stable and maintain their dual ACE and DPP-IV inhibitory activities even after exposure to pepsin, trypsin, and chymotrypsin [21]. Once they reach the small intestine, these stable oligopeptides are primarily absorbed via the PepT1 (Peptide Transporter 1) system. This active transport mechanism is more efficient and rapid than the absorption of free amino acids, ensuring that the bioactive sequences reach the systemic circulation in a form capable of exerting metabolic effects [24,49].

Besides systemic biochemical safety, the effect of SDOs on the gastrointestinal ecosystem, the main site of peptide interaction, is an important aspect of human safety. Following an 8-week intervention, SDOs administration did not significantly alter the composition or relative abundance of the gut microbiota at the family, genus, or species levels, suggesting a favorable gastrointestinal safety profile with no evidence of induced dysbiosis. Following the exclusion of rare OTUs, the gut microbiota of the three experiment groups was made up of seven phyla. This phylum-level profile aligns closely with the seven phyla identified by La-Ongkham et al. (2020) in healthy Thai volunteers [50], with the exception of the phylum *Synergistetes*, which was undetectable in the present study. However, distinct structural variations in relative abundance were observed between the two studies. While La-Ongkham et al. reported Firmicutes as the dominant phylum (mean relative abundance of 67.96% and 63.84%) followed by Bacteroidetes (11.13% and 15.69%) in adult and elderly subjects, respectively, our study observed a dominance of Bacteroidetes (median relative abundance of 50–56%) followed by Firmicutes (median: 32–38%) yielding a Firmicutes-to-Bacteroidetes ratio of approximately 0.6 compared to their reported 7.3-fold. Additionally, the median relative abundance of Actinobacteria was lower in our study (1%) than the 9.8% reported previously [50]. These discrepancies are likely attributable to differences in the 16S rRNA hypervariable regions targeted (V3–V4 primers in our study versus V6–V8 in the previous study), as V3–V4 primers have been documented to provide superior resolution for specific *Bacteroidota* taxa [51,52], though geographical and cohort-specific baseline factors may also contribute. Crucially, despite these initial variations, the stability of the core microbiome over the 8-week period suggests that SDOs do not disrupt microbial homeostasis.

At the family level, our study identified Bacteroidaceae, Enterobacteriaceae, Lachnospiraceae, Oscillospiraceae, Streptococcaceae and Tannerellaceae across all samples, whereas La-ongkham et al. (2020) [50] reported that Lachnospi-raceae and Ruminococcaceae collectively accounted for over 50% of the detected families, with Ruminococcaceae remaining undetected in our participants.

At the genus level, *Phocaeicola* exhibited the highest median relative abundance (10–21%) in our cohort, followed by *Bacteroides* (5–9%) and *Faecalibacterium* (3–7%), contrasting with the reference study, where *Faecalibacterium* was predominant at 15.87% [50]. Using a strict prevalence threshold of 90% or greater, we identified 22 core species, whereas the La-Ongkham study identified 13 core species, with only four (*Blautia luti*, *Blautia wexlerae*, *Faecalibacterium prausnitzii*, and [*Ruminococcus*] *torques*) shared between both studies. Furthermore, *Phocaeicola vulgatus* was the most abundant species in our study (median: 6–13%) but was not part of the core species in the reference cohort, while *F. prausnitzii* accounted for 3–7% in our study compared to 16.7% in theirs. Potential pathogenic signals also remained minimal; while the reference study reported a 6.3–9.7% combined abundance of *Escherichia coli* and *Klebsiella pneumoniae*, *E. coli* was undetectable in our participants, and *K. pneumoniae* was maintained at a minor median relative abundance below 0.3%.

Several beneficial and probiotic taxa were identified within our cohort, supporting baseline intestinal health. Species such as *Bifidobacterium adolescentis* and *B. catenulatum* are typical inhabitants of the adult intestinal tract, while *F. prausnitzii*, an established biomarker for a healthy gut in the Thai population, was also detected in a high proportion in the present study. Common probiotic species include *Lactobacillus* (*L. acidophilus* and *L. casei*) and *Bifidobacterium* (*B. bifidum*) species [53]. In this study, *L. rogosae* and *B. adolescentis* were among the top 20 most abundant species, with median relative abundances of 0.3–0.7%, whereas *Bifidobacterium bifidum* accounted for less than 0.1% of total abundance. The presence of *B. adolescentis* is highly consistent with healthy human profiles, where its prevalence typically ranges between 60% and 80% [54]. Additionally, *L. rogosae* is recognized for its ability to deconjugate bile acids through bile salt hydrolase activity, a function linked to bile metabolism, local detoxification, and cholesterol reduction [55]. On an international scale, our findings align broadly with historical patterns observed by Nam et al. (2011) in South Korea, where *Faecalibacterium*, *Prevotella*, and *Bacteroides* constituted 30.2% of the total microbiota [56]; these three genera were also among our top five most abundant taxa (10–24% combined), though *Phocaeicola* uniquely remained the dominant genus in our study group. No statistically significant differences in the relative abundance of fecal bacteria were observed between placebo, 0.9 g/day SDOs and 1.8 g/day SDOs groups after eight weeks at the family, genus and species taxonomic ranks for the intervention.

While dietary intake was not rigidly standardized through daily recording, participants maintained their habitual diets, which were assessed at baseline to establish a longitudinal reference. This approach was intentionally selected to minimize recording fatigue and response bias, which often lead to declining data quality in multi-week studies [57]. Furthermore, evaluating SDOs within a free-living setting enhances the clinical translatability of our findings, reflecting the safety of the product under actual ‘conditions of use’ as recommended by the European Food Safety Authority (EFSA) guidance [58]. The fact that gut microbial homeostasis was maintained across 41 individuals, each consuming a unique habitual diet, provides evidence that SDOs supplementation does not disrupt the core microbial structure even amidst background dietary variation. While this design may obscure subtle, treatment-specific shifts, it confirms that SDOs are well-tolerated within a real-world dietary context.

Although the study population was 75.6% female, this does not compromise the safety findings. Females often report adverse events more frequently than males [59], suggesting our cohort may provide a sensitive assessment of tolerability. Since biological responses are generally consistent across genders and other factors like BMI and gut microbiota remained stable, these results offer a safety baseline for healthy adults.

The present investigation was designed as a Phase 0 exploratory clinical trial, intended to establish an initial human safety baseline and evaluate early biological impacts with limited human exposure. The sample size of 14 participants per group aligns with established standards for Phase 0 trials, which typically involve 10 to 15 participants to demonstrate proof-of-concept and initial tolerability [60,61,62]. While this cohort size is robust for ensuring that SDOs do not perturb metabolic homeostasis, we acknowledge that it is a limitation of the study that the sample size is not powered to detect rare or uncommon adverse events. In addition, the 8-week duration, although adequate for short-term safety, does not provide data on long-term consumption.

Nevertheless, the safety profile of silkworm-derived proteins is further supported by their application in high-stakes human consumption models, such as aerospace research. The Japan Aerospace Exploration Agency (JAXA) has extensively researched sericin and silkworm pupae as a sustainable ‘space protein’ for use in astronaut food and ecological life support systems [63,64]. Moreover, previous research has explored the incorporation of sericin into various food systems, such as bread, demonstrating its potential as a functional ingredient that enhances nutritional value while remaining effective for human digestion and absorption [65]. These precedents, combined with our clinical findings, fulfill the ‘safety platform’ requirements outlined in the EFSA guidance for a tiered toxicological approach [58]. By confirming that SDOs are well-tolerated at the systemic and microbial levels, this study provides the necessary preliminary evidence to support future larger-scale efficacy trials where uncommon events can be more broadly monitored in diverse populations.

## 5. Conclusions

In conclusion, this exploratory Phase 0 randomized, double-blind, placebo-controlled trial provides preliminary clinical evidence on the short-term safety and tolerability of SDOs produced by enzymatic hydrolysis from *Bombyx mori* yellow silk cocoons. The results showed that short-term oral supplementation up to 1.8 g/day over an 8-week period is well-tolerated by healthy adults, with no serious adverse events reported and only minor transient gastrointestinal symptoms. Safety monitoring via comprehensive clinical testing revealed no clinically significant alterations in hematological parameters or renal and hepatic function markers, suggesting systemic safety following absorption. Furthermore, high-resolution 16S rRNA sequencing suggests that SDOs supplementation preserved gut microbial homeostasis over the 8-week period, supporting its short-term safety at the level of the gastrointestinal microbiome. These findings provide a necessary safety platform for the potential application of SDOs as a novel food ingredient. The preliminary evidence of tolerability for this unique serine and aspartic acid-rich peptide preparation in humans provides a foundation for the next phase of clinical research. Building upon this Phase 0 evidence, the next step in our clinical evaluation will be a Phase 1 trial. This will pave the way for the larger-scale and long-term studies required to evaluate chronic safety and clinical efficacy in diverse or metabolically compromised populations, such as those with pre-hypertension, mild dyslipidemia, or metabolic syndrome.

## Figures and Tables

**Table 1:** Comparative analysis of SDOs and conventional silk peptides.

Feature	SDOs	Conventional Silk Peptides (Hwang et al. [18])
**Raw Material Source**	Pure sericin (yellow silk cocoons)	Whole silk (Mixture of Fibroin and Sericin)
**Production Method**	Controlled enzymatic hydrolysis (Protease N, 55 °C, pH 7.0, 4 h)	Intensive acid hydrolysis (2.5 N HCl, 115 °C, 24 h)
**Top 3 Amino Acids**	Serine (20.5%), aspartic acid (16.9%), glycine (5.2%)	Glycine (31.3%), alanine (20.1%), serine (15.4%)
**Peptide Profile**	Preserves specific bioactive sequences via mild processing	Random mixture of 18 amino acids and small peptides
**Primary Bioactivity**	Metabolic health (antihypertensive, antidiabetic, neuroprotective)	Immunostimulatory (NK cell activity, cytokine production)

**Table 2:** Trace metals and microbial contamination in SDOs powder.

Trace Metal (ppm)		Microbiological Test		
Arsenic (As)	0.105	Coliforms	<3.0	MPN/g
Cadmium (Cd)	No detection	*Escherichia coli*	<3.0	MPN/g
Lead (Pb)	0.299	*Listeria monocytogenes*	negative	per 25 g
Mercury (Hg)	No detection	*Salmonella* spp.	negative	per 25 g
		*Staphylococcus aureus*	<10 cfu/g	
		Total plate count	3.0 × 10^3^ cfu/g	
		Yeasts and molds	<10 cfu/g	

**Table 3:** Amino acid composition of the SDOs powder used in this study.

Amino Acid	SDOs (g/100 g Dry Powder)
**Non-Essential Amino Acids**	
Serine	20.50
Aspartic acid	16.89
Glutamic acid	4.90
Glycine	5.22
Arginine	3.72
Alanine	2.30
Glutamine	2.30
Proline	0.82
Cysteine	0.98
Tyrosine	3.29
**Essential Amino Acids**	
Threonine	5.20
Lysine	5.01
Valine	2.86
Leucine	2.40
Histidine	1.15
Isoleucine	0.71
Phenylalanine	0.74
Methionine	0.41
**Not Detected**	
Asparagine	ND
Tryptophan	ND
**Total Amino Acids**	79.40

Note: ND, not detected.

**Table 4:** Moisture content and water activity (aW) of SDOs powder during 6-month storage at different temperatures.

Temperature (°C)	Time (Month)	Moisture Content (%)	Water Activity (aW)
**4**	0	2.64 ± 0.03 ^a^	0.409 ± 0.01 ^a^
	3	2.67 ± 0.02 ^a^	0.409 ± 0.02 ^a^
	6	2.34 ± 0.15 ^b^	0.382 ± 0.02 ^b^
**25**	0	2.64 ± 0.03 ^a^	0.409 ± 0.01 ^a^
	3	2.24 ± 0.12 ^b^	0.420 ± 0.01 ^a^
	6	2.24 ± 0.14 ^b^	0.370 ± 0.02 ^b^
**45**	0	2.64 ± 0.03 ^a^	0.409 ± 0.01 ^a^
	3	2.23 ± 0.27 ^b^	0.419 ± 0.01 ^a^
	6	2.13 ± 0.15 ^b^	0.373 ± 0.01 ^b^

Data are presented as mean ± standard deviation (SD). Within each temperature condition (4 °C, 25 °C, and 45 °C), values in the same column with different superscript letters (a, b) are significantly different (*p* < 0.05).

**Table 5:** Color parameters of SDOs powder during 6-month storage at different temperatures.

Temp. (°C)	Time (Month)	L* (Lightness)	a* (Redness)	b* (Yellowness)	ΔE (Total Color Difference)
**4**	0	65.34 ± 0.19 ^a^	4.92 ± 0.05 ^a^	26.26 ± 0.11 ^b^	0.00
	3	63.46 ± 0.44 ^b^	4.70 ± 0.03 ^b^	25.40 ± 0.27 ^c^	2.08
	6	58.13 ± 0.26 ^c^	4.14 ± 0.01 ^c^	26.84 ± 0.08 ^a^	7.28
**25**	0	65.34 ± 0.19 ^a^	4.92 ± 0.05 ^a^	26.26 ± 0.11 ^b^	0.00
	3	63.59 ± 0.53 ^b^	4.30 ± 0.06 ^c^	24.16 ± 0.19 ^c^	2.80
	6	58.01 ± 0.45 ^c^	4.62 ± 0.12 ^b^	30.35 ± 0.29 ^a^	8.39
**45**	0	65.34 ± 0.19 ^a^	4.92 ± 0.05 ^b^	26.26 ± 0.11 ^b^	0.00
	3	64.67 ± 0.35 ^b^	3.71 ± 0.04 ^c^	21.30 ± 0.27 ^c^	5.15
	6	57.58 ± 0.17 ^c^	5.35 ± 0.04 ^a^	33.19 ± 0.21 ^a^	10.41

Data are presented as mean ± SD. Within each temperature condition (4 °C, 25 °C, and 45 °C), values in the same column with different superscript letters (a–c) are significantly different (*p* < 0.05). ΔE represents the total color difference calculated from the baseline (Time 0).

**Table 6:** Mean particle diameter and solubility of SDOs powder during 6-month storage.

Temp. (°C)	Time (Month)	Mean Particle Diameter (µm)	Solubility (%)
**4**	0	63.73 ± 0.69	96.94 ± 0.11 ^a^
	3	67.20 ± 1.52	95.75 ± 0.07 ^a^
	6	61.67 ± 4.17	95.80 ± 0.57 ^a^
**25**	0	63.73 ± 0.69	96.94 ± 0.11 ^a^
	3	59.52 ± 0.61	96.48 ± 0.00 ^a^
	6	62.08 ± 4.32	93.88 ± 0.12 ^b^
**45**	0	63.73 ± 0.69	96.94 ± 0.11 ^a^
	3	62.53 ± 4.87	95.11 ± 0.76 ^b^
	6	60.91 ± 2.47	89.70 ± 0.30 ^c^

Data are presented as mean ± SD. No significant differences (*p* > 0.05) were observed for mean particle diameter over time in any condition (4 °C, 25 °C, and 45 °C). For solubility, values in the same column within each temperature condition with different superscript letters (a–c) are significantly different (*p* < 0.05).

**Table 7:** Stability of DPP-IV and ACE inhibitory activities of SDOs powder during 6-month storage.

Temp. (°C)	Time (Month)	DPP-IV Inhibition (IC_50_, mg/mL)	ACE Inhibition (IC_50_, µg/mL)
**4**	0	7.76 ± 0.30 ^b^	28.44 ± 0.39 ^a^
	3	7.93 ± 0.17 ^b^	28.36 ± 0.10 ^a^
	6	9.76 ± 0.52 ^a^	28.63 ± 0.11 ^a^
**25**	0	7.76 ± 0.30 ^b^	28.44 ± 0.39 ^a^
	3	8.13 ± 0.14 ^b^	28.98 ± 0.22 ^a^
	6	9.86 ± 0.53 ^a^	28.62 ± 0.17 ^a^
**45**	0	7.76 ± 0.30 ^b^	28.44 ± 0.39 ^b^
	3	8.16 ± 0.18 ^b^	28.62 ± 0.19 ^b^
	6	10.89 ± 0.24 ^a^	29.40 ± 0.83 ^a^

Data are presented as mean ± SD. IC50 values are expressed on a dry powder basis. Within each temperature condition (4 °C, 25 °C, and 45 °C), values in the same column with different superscript letters (a, b) are significantly different (*p* < 0.05).

**Table 8:** Baseline demographic and physical characteristics of the study participants.

Characteristic	Placebo (n = 14)	SDOs 0.9 g (n = 14)	SDOs 1.8 g (n = 13)	Total (n = 41)	*p*-Value
**Age (years)**	32.5 ± 6.8	27.5 ± 5.3	32.0 ± 6.9	30.6 ± 6.6	0.087
**Sex, n (%)**					0.153 ^1^
Female	13 (92.9)	10 (71.4)	8 (61.5)	31 (75.6)	
Male	1 (7.1)	4 (28.6)	5 (38.5)	10 (24.4)	
**Weight (kg)**	56.6 ± 8.8	60.2 ± 8.4	57.4 ± 9.2	58.1 ± 8.7	0.538
**Height (cm)**	161.3 ± 7.4	165.0 ± 8.5	163.0 ± 7.5	163.1 ± 7.8	0.460
**BMI (kg/m ^2^)**	21.7 ± 2.2	22.0 ± 2.1	21.5 ± 1.9	21.7 ± 2.0	0.815

Data are presented as mean ± SD for continuous variables and as number (n) and percentage (%) for categorical variables. *p*-values were calculated using one-way ANOVA for continuous variables and Fisher’s exact test for categorical variables. 1 *p*-value for the comparison of sex distribution across the three groups.

**Table 9:** Vital signs and hepatic and renal function of participants at baseline, week 4, and week 8.

Parameters	Week	Placebo (n = 14)	SDOs 0.9 g (n = 14)	SDOs 1.8 g (n = 13)	*p*-Value (Interaction)	*p*-Value (Group)	*p*-Value (Time)
**Systolic BP**	0	120.5 ± 12.1	118.3 ± 11.3	118.2 ± 10.0			
**(mm Hg)**	4	116.1 ± 11.4	118.8 ± 13.2	113.8 ± 11.8	0.636	0.448	0.072
	8	112.8 ± 13.9	108.6 ± 8.7	113.2 ± 10.6			
**Diastolic BP**	0	71.3 ± 8.5	68.9 ± 5.3	72.6 ± 10.0			
**(mm Hg)**	4	72.3 ± 11.5	69.6 ± 8.9	68.7 ± 7.6	0.331	0.313	0.386
	8	71.5 ± 12.4	65.4 ± 7.9	69.8 ± 9.5			
**Heart Rate**	0	84.2 ± 12.1	79.4 ± 12.1	78.8 ± 14.1			
**(beats/min)**	4	75.9 ± 12.2	75.1 ± 10.5	76.3 ± 13.4	0.023 *	0.735	0.560
	8	81.6 ± 9.2	73.0 ± 9.2	74.8 ± 10.8			
**Creatinine**	0	0.71 ± 0.13	0.75 ± 0.16	0.76 ± 0.14			
**(mg/dL)**	4	0.72 ± 0.13	0.74 ± 0.16	0.80 ± 0.16	0.438	0.286	0.266
	8	0.74 ± 0.14	0.76 ± 0.17	0.79 ± 0.18			
**BUN (mg/dL)**	0	11.31 ± 2.48	11.64 ± 1.93	11.95 ± 3.29			
	4	11.12 ± 2.71	10.29 ± 2.15	11.57 ± 3.08	0.363	0.592	0.240
	8	10.61 ± 2.46	10.52 ± 2.48	11.05 ± 2.47			
**Total Bilirubin**	0	0.59 ± 0.12	0.47 ± 0.14	0.51 ± 0.15			
**(mg/dL)**	4	0.54 ± 0.19	0.44 ± 0.16	0.40 ± 0.14	0.379	0.384	0.829
	8	0.48 ± 0.20	0.46 ± 0.21	0.41 ± 0.16			
**SGOT (AST)**	0	20.7 ± 7.8	20.9 ± 5.3	22.5 ± 7.9			
**(U/L)**	4	21.9 ± 5.3	22.5 ± 5.5	21.5 ± 5.5	0.146	0.674	0.597
	8	21.2 ± 7.6	18.6 ± 4.1	24.0 ± 11.4			
**SGPT (ALT)**	0	17.9 ± 14.6	19.9 ± 11.7	22.1 ± 18.9			
**(U/L)**	4	18.1 ± 10.6	23.1 ± 14.2	18.3 ± 11.2	0.207	0.911	0.192
	8	23.6 ± 21.2	20.0 ± 11.6	27.4 ± 26.0			
**ALP (U/L)**	0	58.79 ± 22.2	59.71 ± 9.5	62.00 ± 19.5			
	4	63.21 ± 22.1	62.36 ± 9.1	64.15 ± 16.4	0.788	0.753	0.778
	8	63.64 ± 33.6	60.07 ± 11.0	64.46 ± 17.3			

Data are presented as mean ± SD. *p*-values represent the interaction effects and the main effects from linear mixed-effects model analyses. BP = blood pressure; BUN, blood urea nitrogen; AST, aspartate aminotransferase; ALT, alanine aminotransferase; ALP, alkaline phosphatase. *, *p* < 0.05.

**Table 10:** Hematological and metabolic profiles of participants at baseline, week 4, and week 8.

Parameters	Week	Placebo (n = 14)	SDOs 0.9 g (n = 14)	SDOs 1.8 g (n = 13)	*p*-Value (Interaction)	*p*-Value (Group)	*p*-Value (Time)
**WBC**	0	6.43 ± 1.50	6.19 ± 1.05	6.59 ± 1.38			
**(×10^3^ cells/µL)**	4	6.11 ± 1.35	6.40 ± 0.88	6.40 ± 1.45	0.264	0.541	0.090
	8	6.19 ± 1.72	6.03 ± 0.56	5.95 ± 1.47			
**RBC**	0	4.79 ± 0.55	4.77 ± 0.65	4.86 ± 0.57			
**(×10^6^ cells/µL)**	4	4.90 ± 0.61	4.48 ± 0.59	4.81 ± 0.50	0.080	0.264	0.093
	8	4.86 ± 0.55	4.70 ± 0.55	4.84 ± 0.47			
**Hemoglobin**	0	13.3 ± 0.9	12.9 ± 1.5	12.8 ± 1.6			
**(g/dL)**	4	12.9 ± 2.9	13.1 ± 1.6	13.1 ± 1.5	0.281	0.623	0.966
	8	13.5 ± 1.0	12.6 ± 1.6	13.1 ± 1.6			
**Hematocrit (%)**	0	38.7 ± 2.6	38.1 ± 3.7	37.9 ± 4.4			
	4	40.0 ± 3.0	39.2 ± 4.0	38.6 ± 3.9	0.035 *	0.491	0.136
	8	39.6 ± 2.7	38.0 ± 3.6	39.0 ± 4.1			
**Platelets**	0	255.3 ± 42.1	258.4 ± 42.0	252.6 ± 49.6			
**(×10^3^ cells/µL)**	4	263.6 ± 43.5	285.1 ± 44.6	262.8 ± 52.2	0.320	0.341	0.122
	8	265.0 ± 40.5	273.1 ± 36.8	256.2 ± 46.7			
**FBS (mg/dL)**	0	91.6 ± 7.7	90.6 ± 3.8	92.9 ± 7.8	0.870	0.985	0.003 **
	4	88.9 ± 5.7	88.6 ± 6.0	89.0 ± 8.3			
	8	86.3 ± 5.6	85.4 ± 4.4	86.9 ± 8.7			
**TC (mg/dL)**	0	181.0 ± 29.6	189.5 ± 39.6	184.8 ± 45.7	0.561	0.803	0.855
	4	182.9 ± 34.0	186.6 ± 36.9	186.8 ± 52.1			
	8	188.2 ± 35.8	186.6 ± 39.3	183.3 ± 45.6			
**Triglycerides**	0	63.3 ± 15.4	77.2 ± 31.4	105.4 ± 94.2	0.359	0.705	0.855
**(mg/dL)**	4	63.8 ± 25.1	81.2 ± 40.0	105.5 ± 128.0			
	8	76.5 ± 29.8	74.6 ± 35.8	102.6 ± 84.6			
**HDL-C**	0	69.4 ± 18.9	63.7 ± 14.8	60.3 ± 16.3	0.714	0.316	0.176
**(mg/dL)**	4	71.8 ± 15.2	62.7 ± 9.7	62.9 ± 16.7			
	8	68.9 ± 15.7	62.6 ± 11.3	61.3 ± 17.0			
**LDL-C (mg/dL)**	0	99.0 ± 21.4	110.3 ± 34.2	103.6 ± 33.4	0.589	0.956	0.462
	4	98.2 ± 25.5	107.4 ± 31.9	103.2 ± 31.2			
	8	104.1 ± 26.2	109.4 ± 34.1	101.7 ± 28.9			

Data are presented as mean ± SD. WBC, white blood cell; RBC, red blood cell; Hb, hemoglobin; Hct, hematocrit; FBS, fasting blood sugar; TC, total cholesterol. *, *p* < 0.05; **, *p* < 0.01.

**Table 11:** Markers of oxidative stress and inflammation at baseline, week 4, and week 8.

Variable	Week	Placebo (n = 14)	SDOs 0.9 g (n = 14)	SDOs 1.8 g (n = 13)	*p*-Value (Interaction)	*p*-Value (Group)	*p*-Value (Time)
**TAC**	0	159.1 ± 16.1	162.5 ± 14.9	161.2 ± 11.1	0.818	0.750	<0.001 ***
**(TEC µM)**	4	166.8 ± 12.1	166.3 ± 15.4	169.3 ± 10.8			
	8	148.3 ± 10.6	147.9 ± 13.6	150.5 ± 9.5			
**SOD activity (%)**	0	57.2 ± 12.7	62.9 ± 5.5	64.9 ± 8.6	0.187	0.245	0.005 **
	4	82.0 ± 13.5	79.9 ± 11.3	89.2 ± 14.6			
	8	84.6 ± 9.1	83.2 ± 7.6	83.9 ± 22.2			
**MDA Adduct**	0	274.9 ± 115.7	331.6 ± 184.0	209.7 ± 115.1	0.094	0.333	<0.001 ***
**(nM)**	4	392.6 ± 234.7	402.8 ± 175.1	251.6 ± 141.2			
	8	63.8 ± 41.0	70.4 ± 28.2	45.0 ± 29.3			
**hs-CRP (mg/L)**	0	2.30 ± 2.39 (7)	2.45 ± 2.12 (8)	1.85 ± 2.12 (9)	0.759	0.563	0.661
	4	1.39 ± 0.97 (9)	2.36 ± 3.59 (9)	1.23 ± 0.60 (12)			
	8	1.69 ± 1.16 (8)	1.93 ± 2.35 (11)	1.78 ± 1.46 (10)			

Data are presented as mean ± SD. For hs-CRP, data are presented as mean ± SD for quantifiable samples, with the number of quantifiable samples shown in parentheses. *p*-values for the interaction effect and main effect from LMM analyses. TAC, total antioxidant capacity; TEC, Trolox equivalent concentration; SOD, superoxide dismutase; hs-CRP, high-sensitivity C-reactive protein; MDA, malondialdehyde. **, *p* < 0.01; ***, *p* < 0.001.

**Table 12:** Number of participants reporting treatment-emergent adverse events.

Adverse Event	Placebo (n = 14) n (%)	SDOs 0.9 g (n = 14) n (%)	SDOs 1.8 g (n = 13) n (%)
**Gastrointestinal Disorders**			
Bloating or Abdominal Distension	2 (14.3)	5 (35.7)	1 (7.7)
Diarrhea	0 (0.0)	3 (21.4)	2 (15.4)
Constipation	2 (14.3)	2 (14.3)	0 (0.0)
Abdominal Pain	0 (0.0)	1 (7.1)	1 (7.7)
Heartburn	0 (0.0)	0 (0.0)	1 (7.7)
**Nervous System Disorders**			
Headache (including migraine)	5 (35.7)	4 (28.6)	6 (46.2)
Dizziness	1 (7.1)	0 (0.0)	1 (7.7)
Fainting	1 (7.1)	0 (0.0)	0 (0.0)
**Respiratory Disorders**			
Rhinorrhea (with/without sneezing)	2 (14.3)	3 (21.4)	1 (7.7)
Nasal Congestion	2 (14.3)	1 (7.1)	0 (0.0)
Sinusitis	0 (0.0)	1 (7.1)	0 (0.0)
**General Disorders**			
Fever	0 (0.0)	3 (21.4)	0 (0.0)
Weight Gain	1 (7.1)	1 (7.1)	0 (0.0)
Insomnia	1 (7.1)	0 (0.0)	0 (0.0)
**Skin Disorders**			
Itching Rash	1 (7.1)	0 (0.0)	0 (0.0)
Dry Skin	1 (7.1)	0 (0.0)	0 (0.0)
Harden Sebaceous Gland	1 (7.1)	0 (0.0)	0 (0.0)
**Musculoskeletal Disorders**			
Back Pain	1 (7.1)	0 (0.0)	0 (0.0)
Muscle Pain	1 (7.1)	1 (7.1)	0 (0.0)

Data are presented as the number of participants (n) and percentage (%) reporting at least one occurrence of the event.

**Table 13:** Top gut microbiota classified by phylum, class, order, family and genus levels.

Rank	Taxonomy	Percentage Median Relative Abundance (IQR)					
		Placebo		SDOs 0.9 g		SDOs 1.8 g	
		Week 0	Week 8	Week 0	Week 8	Week 0	Week 8
**Phylum**							
**1**	Bacteroidetes	54.85 (11.79)	55.47 (14.93)	55.13 (22.04)	53.04 (24.27)	49.69 (10.60)	55.71 (14.67)
**2**	Firmicutes	33.30 (13.94)	31.20 (13.94)	37.05 (13.19)	37.73 (23.08)	37.10 (18.09)	32.40 (9.78)
**3**	Proteobacteria	4.12 (4.60)	5.83 (4.18)	5.41 (2.49)	4.38 (3.67)	5.21 (2.94)	5.72 (6.97)
**4**	Actinobacteria	1.28 (1.00)	0.97 (1.10)	0.95 (2.26)	0.84 (1.16)	0.83 (3.81)	0.94 (5.38)
**Class**							
**1**	Bacteroidia	54.85 (11.79)	55.47 (14.93)	55.13 (22.04)	53.04 (24.27)	49.69 (10.60)	55.71 (14.67)
**2**	Clostridia	23.08 (9.94)	21.31 (13.64)	20.86 (10.26)	27.82 (17.04)	21.02 (6.48)	16.89 (18.17)
**3**	Negativicutes	8.14 (8.03)	6.76 (7.83)	10.33 (8.21)	4.04 (3.49)	6.33 (8.27)	4.33 (6.27)
**Order**							
**1**	Bacteroidales	54.85 (11.79)	55.47 (14.93)	55.13 (22.04)	53.04 (24.27)	49.69 (10.60)	55.71 (14.67)
**2**	Eubacteriales	23.08 (9.94)	21.31 (13.64)	20.86 (10.26)	27.82 (17.04)	21.02 (6.48)	16.89 (18.17)
**3**	Acidaminococcales	5.28 (7.97)	2.63 (5.02)	8.16 (8.28)	2.69 (4.45)	5.12 (11.82)	2.31 (4.26)
**Family**							
**1**	Bacteroidaceae	20.98 (30.47)	31.79 (32.99)	20.90 (33.53)	27.03 (24.40)	23.46 (22.17)	25.74 (39.38)
**2**	Oscillospiraceae	9.09 (5.41)	10.73 (10.65)	7.31 (5.74)	13.01 (14.98)	7.73 (9.59)	5.93 (11.10)
**3**	Lachnospiraceae	7.27 (5.21)	7.21 (7.05)	9.57 (5.38)	9.77 (3.23)	8.37 (5.45)	6.27 (4.39)
**4**	Acidaminococcaceae	5.28 (7.97)	2.63 (5.02)	8.16 (8.28)	2.69 (4.45)	5.12 (11.82)	2.31 (4.26)
**Genus**							
**1**	*Phocaeicola*	10.79 (19.36)	21.18 (21.89)	17.09 (30.51)	15.58 (21.63)	16.81 (13.25)	10.46 (24.25)
**2**	*Bacteroides*	4.88 (12.41)	7.63 (11.11)	6.69 (9.60)	4.98 (7.52)	7.14 (7.09)	9.41 (8.99)
**3**	*Faecalibacterium*	5.16 (6.20)	4.60 (8.49)	4.83 (5.18)	6.52 (5.86)	2.72 (3.03)	3.39 (5.53)
**4**	*Phascolarctobacterium*	5.27 (9.74)	2.63 (4.80)	7.59 (8.28)	2.67 (4.53)	5.12 (12.72)	2.31 (4.27)
**5**	*Prevotella*	14.44 (50.93)	0.48 (31.73)	0.01 (56.17)	0.50 (57.96)	0.14 (24.85)	0.27 (18.40)
**6**	*Alistipes*	1.35 (2.68)	0.85 (3.79)	0.70 (1.59)	2.78 (4.33)	3.70 (4.40)	3.92 (12.07)
**7**	*Bifidobacterium*	1.14 (1.00)	0.89 (1.00)	0.78 (2.30)	0.47 (1.28)	0.75 (3.77)	0.88 (5.22)

**Table 14:** Top 20 gut microbiota species.

Rank	Phylum	Species	Percentage Median Relative OTU (IQR)					
			Placebo		SDOs900		SDOs1800	
			Baseline	Week 8	Baseline	Week 8	Baseline	Week 8
1	Bacteroidetes	*Phocaeicola vulgatus*	5.80 (8.94)	13.01 (21.60)	7.20 (15.37)	6.20 (12.17)	11.24 (10.59)	8.00 (5.89)
2	Firmicutes	*Faecalibacterium prausnitzii*	5.16 (6.20)	4.60 (8.49)	4.83 (5.18)	6.52 (5.86)	2.72 (3.03)	3.39 (5.53)
3	Firmicutes	*Phascolarctobacterium faecium*	3.27 (10.41)	2.19 (4.37)	7.54 (12.08)	0.97 (4.81)	0.12 (5.73)	0.08 (2.31)
4	Bacteroidetes	*Alistipes putredinis*	0.70 (2.42)	0.11 (2.61)	0.36 (1.21)	1.99 (3.57)	3.11 (3.87)	3.43 (5.13)
5	Bacteroidetes	*Bacteroides uniformis*	0.89 (3.46)	1.65 (4.89)	0.81 (1.93)	1.41 (1.88)	1.10 (2.13)	3.46 (6.49)
6	Firmicutes	[*Eubacterium*] *rectale*	1.13 (1.10)	1.65 (1.98)	0.97 (2.93)	2.02 (2.94)	0.75 (1.77)	0.76 (0.74)
7	Firmicutes	*Oscillibacter ruminantium*	0.66 (0.63)	0.37 (0.68)	0.84 (0.89)	1.48 (2.12)	0.62 (3.64)	1.24 (4.54)
8	Bacteroidetes	*Prevotella copri*	3.86 (43.28)	0.34 (12.74)	0.00 (48.39)	0.06 (44.41)	0.14 (10.96)	0.27 (13.43)
9	Firmicutes	*Blautia wexlerae*	0.51 (0.55)	0.85 (0.71)	0.70 (0.66)	0.54 (0.80)	0.67 (0.43)	0.43 (0.35)
10	Bacteroidetes	*Phocaeicola massiliensis*	0.20 (1.58)	0.03 (0.87)	1.11 (5.78)	2.01 (3.38)	0.14 (2.66)	0.18 (3.14)
11	Bacteroidetes	*Parabacteroides merdae*	0.49 (0.58)	0.46 (1.15)	0.60 (0.93)	0.44 (0.64)	0.95 (0.83)	0.67 (1.20)
12	Firmicutes	*Lachnospira eligens*	0.59 (0.68)	0.58 (1.24)	0.41 (1.25)	0.75 (0.79)	0.49 (0.29)	0.78 (0.88)
13	Bacteroidetes	*Bacteroides thetaiotaomicron*	0.52 (1.08)	0.50 (0.77)	0.43 (0.58)	0.49 (0.44)	0.66 (0.97)	0.83 (0.80)
14	Firmicutes	*Gemmiger formicilis*	0.60 (1.20)	0.44 (1.70)	0.49 (1.20)	0.75 (1.48)	0.37 (1.17)	0.55 (0.82)
15	Actinobacteria	*Bifidobacterium adolescentis*	0.62 (1.08)	0.55 (1.12)	0.63 (2.14)	0.36 (0.98)	0.47 (3.83)	0.49 (1.54)
16	Firmicutes	*Lactobacillus rogosae*	0.35 (0.77)	0.62 (1.04)	0.33 (0.42)	0.36 (0.34)	0.74 (1.64)	0.72 (1.19)
17	Bacteroidetes	*Parabacteroides distasonis*	0.29 (0.37)	0.88 (1.69)	0.45 (0.85)	0.47 (0.64)	0.55 (0.45)	0.36 (0.90)
18	Firmicutes	*Roseburia inulinivorans*	0.55 (1.58)	0.33 (0.42)	0.68 (2.20)	0.49 (0.98)	0.65 (1.81)	0.20 (1.53)
19	Bacteroidetes	*Bacteroides stercoris*	0.38 (0.63)	0.59 (1.04)	0.39 (2.69)	0.82 (2.59)	0.27 (0.56)	0.41 (1.56)
20	Bacteroidetes	*Bacteroides caccae*	0.45 (1.18)	0.23 (0.79)	0.63 (1.90)	0.31 (0.86)	0.97 (1.88)	0.27 (0.32)

**Table 15:** Alpha diversities in placebo, SDOs 0.9 g and SDOs 1.8 g groups at week 0 and 8.

Alpha Diversity Indices	Median Alpha Diversity Indices (IQR)					
	Placebo		SDOs 0.9 g		SDOs 1.8 g	
	Baseline	Week 8	Baseline	Week 8	Baseline	Week 8
Observed OTUs *	90.5 (24.3)	86.0 (30.3)	81.5 (20.5)	94.0 (28.0)	102.0 (19.0)	92.0 (34.0)
Chao 1 *	100.1 (30.4)	96.4 (30.2)	86.1 (26.0)	103.5 (26.6)	109.5 (21.1)	102.0 (37.5)
**Shannon index**	2.8 (0.9)	2.8 (0.8)	2.7 (1.0)	2.7 (1.2)	3.2 (0.6)	2.7 (0.9)
**Inverse Simpson index**	8.2 (6.5)	8.5 (7.0)	8.4 (8.5)	7.6 (11.2)	12.6 (6.5)	7.0 (11.5)

* *p*-value for group by time effects < 0.05 using nparLD.

## Data Availability

The original contributions presented in this study are included in the article. Further inquiries can be directed to the corresponding author.
